# ^177^Lu-Labeled HEDP-Functionalized Manganese-Substituted Maghemite Nanocarriers for Localized Radionuclide Therapy

**DOI:** 10.3390/pharmaceutics18101324

**Published:** 2026-10-19

**Authors:** Dragana Stanković, Magdalena Radović, Pavle Sitarica, Miloš Marić, Aleksandar Vukadinović, Miloš Ognjanović, Marija Mirković, Drina Janković, Mirjana Milić, Bratislav Antić, Sanja Vranješ Đurić

**Affiliations:** VINČA Institute of Nuclear Sciences, National Institute of the Republic of Serbia, University of Belgrade, P.O. Box 522 Belgrade, Serbia; dragana.s@vin.bg.ac.rs (D.S.);

**Keywords:** nanoradiopharmaceuticals, lutetium-177, manganese-substituted maghemite, HEDP, intratumoral delivery, radiochemical stability, magnetic hyperthermia, nanobrachytherapy

## Abstract

**Background/Objectives**: Effective intratumoral radionuclide therapy requires nanocarriers that enable stable radionuclide coordination and prolonged tumor retention. Integrating these properties with efficient magnetic heating within a single nanoplatform remains challenging because optimization of one property may compromise another. This study investigated whether manganese substitution could improve the magnetic heating performance of 1-hydroxyethylidene-1,1-diphosphonic acid (HEDP)-functionalized maghemite nanoparticles while preserving their suitability for radiopharmaceutical applications. **Methods**: Three Mn*_x_*Fe_2−*x*_O_3_ compositions (*x* = 0, 0.221, and 0.480) were evaluated in terms of their structural and physicochemical properties, colloidal stability, and magnetic heating performance. The most promising Mn-substituted formulation and the unsubstituted reference were evaluated by ^177^Lu radiolabeling, in vitro radiochemical stability testing, whole-body imaging, ex vivo biodistribution analysis, and therapeutic efficacy assessment in 4T1 tumor-bearing mice. **Results**: Moderate Mn substitution (*x* = 0.221) provided the most favorable combination of magnetic heating performance (SAR = 360 W g^−1^; ILP = 5.65 nH m^2^ kg^−1^) and colloidal stability. HEDP functionalization enabled direct radiolabeling with ^177^Lu, with labeling yields of 86–92% and radiochemical stability exceeding 94% after 96 h in saline and human serum. Following intratumoral administration, qualitative whole-body imaging showed persistent tumor-associated radioactivity for both nanoparticle formulations on days 5 and 14. On day 14, tumor-associated activity was 91.68 ± 0.73%ID and 84.53 ± 2.26%ID for [^177^Lu]Lu-NP1 and [^177^Lu]Lu-NP2, respectively, compared with 17.49 ± 8.06%ID for free [^177^Lu]LuCl_3_. Both nanoparticle formulations inhibited tumor growth compared with saline, with a more pronounced response and partial tumor regression in animals bearing smaller tumors at treatment initiation. **Conclusions**: Moderate Mn substitution improved magnetic heating performance and colloidal stability while preserving the radiochemical stability, intratumoral retention, and therapeutic efficacy of the ^177^Lu-labeled HEDP-functionalized nanocarriers. This nanoplatform warrants further investigation for intratumoral ^177^Lu therapy and its potential integration with magnetic hyperthermia.

## 1. Introduction

Radiotherapy remains a backbone of solid tumor treatment; however, delivering a sufficiently high radiation dose to the tumor while sparing surrounding healthy tissues remains a major clinical challenge. Conventional brachytherapy addresses this limitation by positioning sealed radioactive sources directly within or adjacent to the tumor, thereby enabling highly localized dose delivery. Despite its clinical success, conventional brachytherapy requires invasive or minimally invasive source placement and relies on accurate implantation to achieve the intended dose distribution [1,2]. Consequently, increasing attention has been directed toward injectable radiolabeled nanocarriers capable of functioning as localized radiation sources following intratumoral administration [1,2,3]. By delivering radiolabeled nanocarriers directly to the tumor, intratumoral administration may promote local radionuclide retention while reducing, although not eliminating, systemic redistribution and exposure of non-target tissues [2,3,4,5,6]. Among the nanomaterials investigated as carriers for localized radionuclide therapy, magnetic iron oxide nanoparticles are particularly attractive because of their favorable biocompatibility and tunable structural, magnetic, and surface properties. Their physicochemical behavior and magnetic response are governed by crystal structure, particle size, morphology, and chemical composition. In particular, multicore nanoflower architectures have attracted considerable attention because the cooperative magnetic behavior arising from the ordered organization of their primary crystallites can enhance magnetic response and heating efficiency under an alternating magnetic field [5,7,8,9]. Magnetic heating may provide an additional therapeutic function and could potentially complement the localized radiation dose delivered by radiolabeled nanoparticles.

Beyond morphology, partial cation substitution provides another strategy for tuning the magnetic properties of spinel iron oxides. Incorporation of transition-metal cations into the iron oxide lattice can modify lattice structure, magnetic anisotropy, and saturation magnetization, thereby influencing heat generation under an alternating magnetic field [7,10,11]. Although cation substitution has been widely studied as a way to improve magnetic heating performance and magnetic resonance imaging performance, its use in nanoparticles designed for localized radionuclide therapy has received much less attention. In these systems, improving one property may adversely affect another. Magnetic heating performance therefore needs to be considered together with colloidal stability, radionuclide coordination, radiochemical stability, biological performance, and intratumoral retention, rather than optimized in isolation [5].

Localized radionuclide therapy requires therapeutic radionuclides capable of delivering a sufficiently high absorbed dose to the tumor, while minimizing irradiation of surrounding healthy tissues [12]. Lutetium-177 (^177^Lu) is particularly suitable for this purpose because of its favorable physical half-life (6.65 d), medium-energy β^−^ emission with a relatively short tissue penetration range (mean 670 μm and maximum 2 mm), and accompanying γ-emissions (6.4% of 113 keV and 11% of 208 keV) that enable post-therapy imaging and dosimetry [12]. These decay characteristics are particularly advantageous for intratumoral radionuclide therapy, provided that the radionuclide is retained at the injection site to sustain local irradiation while minimizing systemic exposure [2,3,4,5,12].

In addition to the properties of the magnetic core, surface chemistry plays a critical role in determining colloidal stability, biological interactions, radionuclide coordination, and radiochemical stability. 1-Hydroxyethylidene-1,1-diphosphonic acid (HEDP) is particularly suitable for this purpose because its phosphonate groups bind strongly to iron oxide surfaces, while its oxygen donor atoms can coordinate lanthanide radionuclides. HEDP can therefore serve both as a surface ligand and as a coordination site for radiometals, potentially eliminating the need for an additional bifunctional chelator and simplifying the formulation [13,14].

Despite these advantages, it remains unclear whether Mn substitution can improve the magnetic heating performance of HEDP-functionalized maghemite nanoparticles while preserving the radiochemical stability and biological performance required for localized radionuclide therapy. Therefore, this study investigated the effects of Mn content on the structural and physicochemical properties, colloidal stability, and magnetic heating performance of Mn*_x_*Fe_2−*x*_O_3_ nanoparticles. Based on these results, the most promising Mn-substituted formulation was selected and compared with unsubstituted maghemite in terms of ^177^Lu-labeling, radiochemical stability, biodistribution, intratumoral retention, and therapeutic efficacy in 4T1 tumor-bearing mice. The relationship between tumor size at treatment initiation and therapeutic response was also examined.

## 2. Materials and Methods

### 2.1. Materials

All chemicals were of analytical grade and were used as received. Iron(III) chloride hexahydrate (FeCl_3_·6H_2_O), iron(II) chloride tetrahydrate (FeCl_2_·4H_2_O), iron(III) nitrate nonahydrate (Fe(NO_3_)_3_·9H_2_O), manganese(II) chloride tetrahydrate (MnCl_2_·4H_2_O), diethylene glycol (DEG), N-methyldiethanolamine (NMDEA), sodium hydroxide (NaOH), sodium chloride (NaCl), ethanol, ethyl acetate, nitric acid (HNO_3_) and 1-hydroxyethylidene-1,1-diphosphonic acid (etidronic acid, HEDP) were purchased from Sigma-Aldrich (St. Louis, MO, USA). Ultrapure Milli-Q water (Millipore Co., Billerica, MA, USA) was used throughout all experiments. No-carrier-added ^177^LuCl_3_ was supplied by ITG Isotope Technologies Garching GmbH (Munich, Germany) with a specific activity > 500 GBq/mg Lu.

### 2.2. Synthesis of Mn_x_Fe_2−x_O_3_ Nanoparticles

The Mn*_x_*Fe_2−*x*_O_3_ nanoparticles, including γ-Fe_2_O_3_, Mn_0.221_Fe_1.779_O_3_ and Mn_0.480_Fe_1.520_O_3_, were synthesized via a modified polyol method [15], with the total metal cation content maintained at 4.8 mmol. The stoichiometric ratios of the metal precursors were initially selected to target nominal Mn substitution levels of *x* = 0.333 and *x* = 0.666. Based on subsequent ICP-OES analysis, the actual compositions were determined to be Mn_0.221_Fe_1.779_O_3_ and Mn_0.480_Fe_1.520_O_3_, respectively, and these experimentally determined compositions are used throughout this manuscript. For the synthesis of Mn*_x_*Fe_2−*x*_O_3_, the required masses of MnCl_2_·4H_2_O (0 g, 0.1582 g, and 0.3163 g, respectively), FeCl_3_·6H_2_O (0.8650 g, 0.7209 g, and 0.5795 g, respectively) and FeCl_2_·4H_2_O (0.3181 g, 0.2656 g and 0.2131 g, respectively) were dissolved in 64 g of a 50:50 (*w*/*w*) mixture of DEG and NMDEA and stirred vigorously for 60 min. Separately, a basic solution was prepared by sonication of 0.512 g of NaOH in 32 g of the same solvent mixture at 80 °C for one hour. Upon complete dissolution, the NaOH solution was added to the metal precursor solution, and the resulting mixture was stirred for an additional 1 h before being transferred to a 100 mL Teflon-lined reactor for solvothermal treatment at 220 °C for 6 h. After oven-cooling, the resulting nanoparticles were magnetically separated and washed with a 50:50 ethanol/ethyl acetate mixture. To ensure complete oxidation, the nanoparticles underwent an acidic treatment with 30 mL of 10% HNO_3_ and were heated to 80 °C with the addition of 6.6 g of Fe(NO_3_)_3_∙9H_2_O. Following a final nitric acid wash and several deionized water rinses, the purified Mn*_x_*Fe_2−*x*_O_3_ nanoparticles were redispersed in Milli-Q water.

### 2.3. HEDP Surface Functionalization of Mn_x_Fe_2−x_O_3_ Nanoparticles

HEDP functionalization of the prepared nanoparticles was performed according to a modified protocol based on the procedure reported by Mirković et al. [14]. HEDP was dissolved in deionized water to prepare a 0.9 M solution. The magnetic nanoparticle suspensions were adjusted to a concentration of 60 mg/mL. Equal volumes of nanoparticle suspension (containing approximately 250 mg) and HEDP solution were mixed at a nanoparticle-to-HEDP molar ratio of 1:2 and the pH of the resulting mixtures was adjusted to 9. The suspension was incubated under gentle stirring for 2 h at 30 °C. The functionalized nanoparticles were purified by overnight dialysis (12 kDa MWCO) against deionized water and stored at 4 °C. Based on the physicochemical characterization, Mn_0.221_Fe_1.779_O_3_@HEDP (NP2), which exhibited the most favorable combination of magnetic and colloidal properties among the Mn-substituted samples, was selected for subsequent radiolabeling and biological evaluation, together with γ-Fe_2_O_3_@HEDP (NP1) as the unsubstituted reference formulation.

### 2.4. Physicochemical Characterization

#### 2.4.1. Elemental and Structural Characterization

To determine the elemental composition of the Mn*_x_*Fe_2−*x*_O_3_ samples, inductively coupled plasma optical emission spectroscopy (ICP-OES) was performed using an iCAP Duo spectrometer (Thermo Fisher Scientific Inc., Waltham, MA, USA). The iron and manganese concentrations were quantified by monitoring the specific emission lines at 238.204 nm and 257.610 nm, respectively.

The crystal structure of the Mn*_x_*Fe_2−*x*_O_3_ nanoparticles was characterized using X-ray powder diffraction (XRPD). Measurements were performed on dried powder samples with a high-resolution Smart Lab^®^ diffractometer (Rigaku, Tokyo, Japan) using *CuKα* radiation (*λ* = 1.5406 Å) at operating parameters of 40 kV and 30 mA. Data were recorded over a 2*θ* range of 10–50° with a scanning speed of 0.5 °/min and a step size of 0.02°. The average crystallite size was calculated via the Scherrer equation using the PDXL2 integrated software suite (Version 2.8.40, Rigaku Corporation, Tokyo, Japan).

#### 2.4.2. Morphological and Surface Characterization

Transmission electron microscopy (TEM) was performed using an FEI Talos F200X microscope (Thermo Fisher Scientific Inc., Waltham, MA, USA) operated at 200 kV. Samples were prepared by depositing dilute aqueous suspensions onto carbon-coated copper grids and allowing them to air dry.

The surface functional groups of the prepared samples were analyzed by Fourier-transform infrared (FTIR) spectroscopy using a Nicolet™ iS50 spectrometer (Thermo Fisher Scientific Inc., Waltham, MA, USA) equipped with an attenuated total reflection (ATR) accessory. Spectra were recorded directly from dried powder samples over the 4000–400 cm^−1^ range with a spectral resolution of 4 cm^−1^.

#### 2.4.3. Colloidal Characterization

The hydrodynamic diameters of bare Mn*_x_*Fe_2−*x*_O_3_ and HEDP-coated Mn*_x_*Fe_2−*x*_O_3_ nanoparticles were determined by dynamic light scattering (DLS) using a NanoZS90 instrument (Malvern Panalytical, Malvern, UK) equipped with a 633 nm He–Ne laser. Measurements were performed on aqueous suspensions (0.1 mg/mL) in deionized water at 25.0 ± 0.1 °C. The zeta potential was measured over the pH range 2–12 using DTS1070 disposable capillary cells. Sample pH was adjusted with dilute HNO_3_ or NaOH to determine the isoelectric point (*pI*).

#### 2.4.4. Magnetic Heating Measurements

Magnetic hyperthermia experiments were performed using a DM100 nB calorimeter (nanoScale Biomagnetics, Zaragoza, Spain). Heating curves of HEDP-coated Mn*_x_*Fe_2−*x*_O_3_ nanoparticles were recorded for 60–200 s using 1 mL aqueous dispersions at a concentration of 4.5 mg/mL. Measurements were carried out under an alternating magnetic field with a frequency of 252 kHz and an amplitude of 200 Oe. Temperature changes were monitored using an optical fiber probe. The specific absorption rate (SAR) was calculated according to Equation (1):

(1) $$SAR=\frac{C}{m_{NP}}{\left(\frac{dT}{dt}\right)}_{t\to 0},$$

where *C* is the total sample heat capacity, *m_NP_* is the mass of nanoparticles, and (*dT/dt)_t→_*_0_ is the initial slope of the heating curve. The intrinsic loss power (ILP) was calculated according to Equation (2):

(2) $$ILP=\frac{SAR}{fH_{0}^{2}},$$

where *f* is the field frequency and *H*_0_ is the magnetic field amplitude.

### 2.5. Radiolabeling of NP1 and NP2 with ^177^Lu

The radiolabeling of the unsubstituted reference formulation, γ-Fe_2_O_3_@HEDP (NP1) and the Mn-substituted formulation, Mn_0.221_Fe_1.779_O_3_@HEDP (NP2), with ^177^Lu was performed following a previously reported procedure [16]. Briefly, 5 μL containing approximately 185 MBq of [^177^Lu]LuCl_3_ was added to separate 0.5 mL aliquots of the aqueous suspensions of NP1 and NP2 (5 mg/mL, pH 4.5). The reaction mixtures were incubated for 1 h at room temperature under continuous stirring. The radiolabeling yields of [^177^Lu]Lu-NP1 and [^177^Lu]Lu-NP2 were determined by radio thin-layer chromatography (radio TLC) using saline as the mobile phase and silica gel (SG) ITLC strips (ITLC-SG, Agilent Technologies, Santa Clara, CA, USA) as the stationary phase. Radio-TLC was carried out using a Scan-RAM scanner (LabLogic Group, Sheffield, South Yorkshire, UK). Under these chromatographic conditions, the labeled nanoparticles remained at the origin (*R_f_* = 0.0–0.1), whereas unbound [^177^Lu]LuCl_3_ migrated with the solvent front (*R_f_* = 0.8–0.9). To verify the reliability of the radio-TLC procedure, radiolabeled nanoparticle samples were centrifuged at 15,000× *g* for 30 min. The radioactivity associated with the precipitate and supernatant was subsequently quantified using a gamma counter (Wizard 2480, PerkinElmer, Shelton, CT, USA).

### 2.6. In Vitro Radiochemical Stability of [^177^Lu]Lu-NP1 and [^177^Lu]Lu-NP2

The radiochemical stability of [^177^Lu]Lu-NP1 and [^177^Lu]Lu-NP2 was assessed by incubating 100 µL of each suspension separately in 1.9 mL of saline and human serum (National Blood Transfusion Institute, Belgrade, Serbia). The release of free ^177^Lu relative to the nanoparticle-bound fraction was monitored during incubation at 37 °C for up to 96 h. Aliquots (50 μL) were withdrawn at 1, 24, 48, and 96 h and analyzed by radio-TLC on SG strips using saline as the mobile phase. The proportion of free ^177^Lu was determined from the chromatographic profiles as a measure of radiochemical stability of [^177^Lu]Lu-NP1 and [^177^Lu]Lu-NP2.

### 2.7. Cell Culture

Murine 4T1 mammary carcinoma cells (ATCC, Manassas, VA, USA; ATCC^®^ CRL-2539™) were thawed from frozen stocks at 37 °C and immediately diluted in 8 mL of cold RPMI-1640 medium. The cells were then centrifuged at 600× *g* for 4 min. The supernatant was discarded, and the cell pellet was resuspended in 12 mL of complete RPMI-1640 medium supplemented with 10% (*v*/*v*) fetal bovine serum (FBS), 100 IU/mL penicillin, and 100 μg/mL streptomycin. Cells were maintained at 37 °C in a humidified atmosphere containing 5% CO_2_. Upon reaching approximately 80% confluence in monolayer culture, the cells were detached using trypsin, washed twice with phosphate-buffered saline (PBS), and resuspended in RPMI-1640 medium. Cell number was determined using a Neubauer hemocytometer, and the cell density was adjusted to a final concentration of 4 × 10^6^ cells/mL.

### 2.8. Establishment of the 4T1 Tumor-Bearing Mouse Model

Experimental animals (female BALB/c mice, 8–10 weeks old) were obtained from the Institute for Biological Research “Siniša Stanković”, Belgrade, Serbia. Animals were housed under controlled conditions in a P/N ventilated IVC cage system (Allentown, PA, USA) at a constant temperature (22 ± 2 °C) and humidity (45–50%). They were maintained under a 12 h/12 h light/dark cycle, with ad libitum access to a standard commercial pellet diet and drinking water. Four mice were housed per polypropylene cage with wood-chip bedding. Prior to experiments, animals were acclimatized for one week to the laboratory environment. All experimental procedures involving animals were approved by the Ethical Committee for care and use of laboratory animals of the Vinča Institute of Nuclear Sciences Belgrade, No. 116-5/2022-000, and by the Ministry of Agriculture and Environmental Protection of the Republic of Serbia (approval No. 323-07-10153/2022-05), in accordance with the National Animal Welfare Law and EU Directive 2010/63/EU. Murine 4T1 breast carcinoma cells were used to establish the tumor model. Each mouse received a single subcutaneous injection of 4 × 10^5^ 4T1 cells suspended in 100 μL RPMI medium into the shaved flank of one hind leg. Tumor growth was monitored every other day. Tumor dimensions were measured with a digital caliper, and volumes were calculated using Equation (3):

*V* (mm^3^) = length × width × height/2 (3)

Experiments were terminated if tumors reached ≥2000 mm^3^ or caused excessive burden, with euthanasia performed by cervical dislocation. Body weight was recorded before dosing and grouping (day 0), and then every other day (immediately before tumor measurement) in both the treated and control groups. At study completion, mean body weight gains were calculated for each group.

### 2.9. Therapeutic Efficacy Study

The therapeutic efficacy of [^177^Lu]Lu-NP1 and [^177^Lu]Lu-NP2 formulations was evaluated in relation to the initial tumor size. On the day of radiolabeled particle injection (day 0), tumors were stratified into two cohorts based on size: small tumors (45–75 mm^3^) and large tumors (150–200 mm^3^). Each cohort was randomly subdivided into four groups (six mice per group), including one control group. Animals received intratumoral injections of saline (50 μL, control), free [^177^Lu]LuCl_3_ (7.4 MBq/50 μL), [^177^Lu]Lu-NP1 (7.4 MBq/200 μg/50 μL) or [^177^Lu]Lu-NP2 (7.4 MBq/200 μg/50 μL). Animal welfare was monitored daily, whereas tumor volume and body weight, used to monitor general treatment-related effects, were recorded every other day for 14 days. Tumor growth kinetics were subsequently analyzed to evaluate the therapeutic efficacy of the different treatment groups in relation to the initial tumor size.

### 2.10. In Vivo Imaging and Ex Vivo Biodistribution Studies

In vivo imaging was performed using a BRUKER^®^ In Vivo Xtreme II imaging system (Billerica, MA, USA). One mouse was randomly selected from each experimental group and used as a representative animal for the imaging study. The imaging experiment was intended to visualize the overall localization of radioactivity at the selected time points and was not used for quantitative or statistical comparisons among groups. The same animals were imaged on days 5 and 14 after intratumoral administration. At each imaging time point, mice were anesthetized by intraperitoneal administration of ketamine/xylazine (90/10 mg/kg) and positioned in the imaging chamber. Images were acquired in the prone position with an exposure time of 2 s using the onboard Xtreme II CCD imaging system.

At the study endpoint (day 14), mice were euthanized by cervical dislocation. Tumors and major organs were excised and weighed, and their radioactivity was quantified using a gamma counter (Wizard 2480, PerkinElmer, Shelton, CT, USA). The biodistribution of radiolabeled compounds was expressed as the percentage of the injected dose per gram of tissue (%ID/g), calculated by comparing the sample activity to that of the standard representing 100% of the injected dose. All data are presented as mean ± standard deviation (SD).

## 3. Results and Discussion

### 3.1. Structural and Compositional Characterization

The precursor solutions were prepared according to nominal Mn substitution levels of *x* = 0, 0.333, and 0.666 (Mn*_x_*Fe_2−*x*_O_3_), whereas the actual Mn contents were determined by ICP-OES to establish reliable composition–structure–property relationships, since the final composition of spinel ferrites cannot be determined solely from the precursor stoichiometry. The measured elemental compositions deviated from the nominal values, yielding empirical formulas of Mn_0.221_Fe_1.779_O_3_ and Mn_0.480_Fe_1.520_O_3_ for the Mn-substituted samples, corresponding to Mn incorporation efficiencies of 66.4% and 72.1%, respectively. Similar discrepancies between nominal precursor composition and experimentally determined Mn contents have been reported previously for Mn-substituted iron oxide nanoparticles, highlighting the importance of correlating material properties with the measured rather than the nominal composition [17,18]. Increasing the nominal Mn substitution level from *x* = 0.333 to *x* = 0.666 increased the experimentally determined Mn content from *x* = 0.221 to *x* = 0.480. However, the incorporated Mn remained below the nominal value at both substitution levels, consistent with previous reports [18]. Because ICP-OES does not provide information on cation valence states, site occupancy, or vacancy distribution, the charge-compensation mechanism cannot be established from the present data. In Mn-containing spinel ferrites, charge balance and cation distribution may involve cation vacancies and mixed Mn/Fe valence states; however, their presence in the present samples would require dedicated spectroscopic or structural confirmation [19].

Despite the deviation from the nominal composition, no secondary crystalline phases were detected by XRPD, and all samples exhibited the characteristic reflections of the spinel structure.

**Figure 1:**
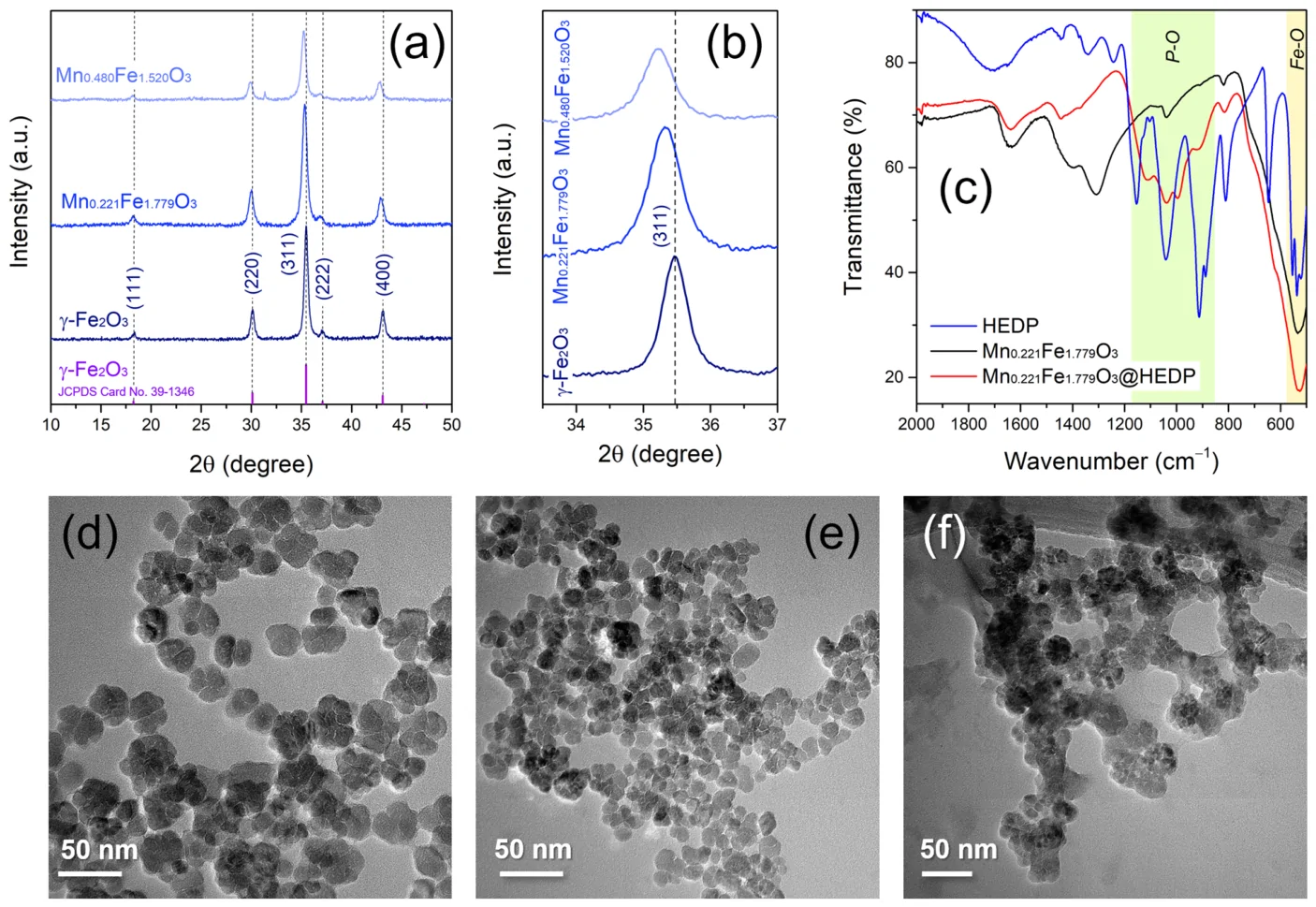
(**a**) XRPD patterns of γ-Fe_2_O_3_, Mn_0.221_Fe_1.779_O_3_, and Mn_0.480_Fe_1.520_O_3_; (**b**) enlarged view of the (311) reflection, (**c**) FTIR spectra of HEDP, Mn_0.221_Fe_1.779_O_3_ and Mn_0.221_Fe_1.779_O_3_@HEDP. (**d**–**f**) TEM micrographs of γ-Fe_2_O_3_, Mn_0.221_Fe_1.779_O_3_ and Mn_0.480_Fe_1.520_O_3,_ respectively.

The XRD patterns of all synthesized Mn-substituted iron oxide nanoparticles are characteristic of a spinel structure (Figure 1a). Because nanocrystalline magnetite (Fe_3_O_4_) and maghemite (γ-Fe_2_O_3_, JCPDS Card No. 39-1346) have closely related crystal structures and lattice parameters, these phases cannot be distinguished reliably by XRD patterns alone. Based on previous studies from our group employing the same polyol synthesis and oxidation procedure, the present samples were assigned to a maghemite-based spinel structure [20]. The diffraction peaks were indexed using the cubic spinel structural model. No additional reflections attributable to secondary iron- or manganese-oxide phases were detected. As the Mn content increased from *x* = 0 to *x* = 0.480, the (311) reflection shifted systematically toward lower 2θ values (Figure 1b). This systematic shift supports Mn incorporation into the spinel lattice and is consistent with lattice expansion, in agreement with previous XRD observations for Mn-substituted iron oxide spinels [21]. Although ICP-OES confirms the overall Mn content, neither ICP-OES nor XRD can establish the oxidation state of manganese. The observed diffraction changes therefore do not identify the Mn oxidation state or its distribution among the available lattice sites. The observed peak broadening is consistent with a decrease in the average crystallite size, which decreased from 20.4 ± 0.8 nm for γ-Fe_2_O_3_ to approximately 15 nm for the Mn-substituted samples, as estimated using the Scherrer equation (Table 1). Collectively, these results indicate that partial Mn substitution is associated with changes in lattice parameters and crystallite size while preserving the spinel framework, thereby providing a common crystalline framework for evaluating composition-dependent changes in the subsequent physicochemical, magnetic, and radiopharmaceutical properties.

TEM analysis revealed multicore, flower-like architectures in all three samples, although the degree of nanoflower organization varied with composition. The γ-Fe_2_O_3_ sample predominantly contained well-developed nanoflower-like particles, together with some particle clustering. In the Mn_0.221_Fe_1.779_O_3_ sample, nanoflower-like structures remained present, together with a larger proportion of smaller and less regularly organized multicore clusters. At the highest Mn content, multicore nanoflower-like units were still evident but showed pronounced association into larger agglomerates. Multiple regions of one TEM grid per sample were examined and showed similar morphological patterns. However, frequent overlap and association of the multicore particles prevented reliable particle-size analysis. Because particle association may also be influenced by deposition and drying on the TEM grid, these observations were interpreted qualitatively.

FTIR spectroscopy confirmed the successful surface functionalization of Mn*_x_*Fe_2−*x*_O_3_ nanoparticles with HEDP ligands (Figure 1c and Figure S1). The FTIR spectra of the HEDP-coated nanoparticles exhibit characteristic phosphonate P–O stretching vibrations in the 900–1150 cm^−1^ region, which are absent in the spectra of bare nanoparticles. The broadening and slight shift in these bands relative to free HEDP are consistent with coordination of the phosphonate groups to surface metal cations, in agreement with the mono- and bidentate Fe–O–P surface coordination modes previously reported for phosphonate-functionalized iron oxide nanoparticles [22]. The persistence of the Fe–O lattice vibration at approximately 545 cm^−1^ in both bare and coated nanoparticles is consistent with preservation of the iron oxide core following surface functionalization. The observations support HEDP surface modification without evidence of major changes to the iron oxide core. The resulting phosphonate-rich surface provides coordination sites for subsequent ^177^Lu-labeling and establishes the surface chemistry underlying the colloidal and radiochemical behavior discussed in the following sections.

### 3.2. Colloidal Properties of Bare and HEDP-Functionalized Mn_x_Fe_2−x_O_3_ Nanoparticles

**Figure 2:**
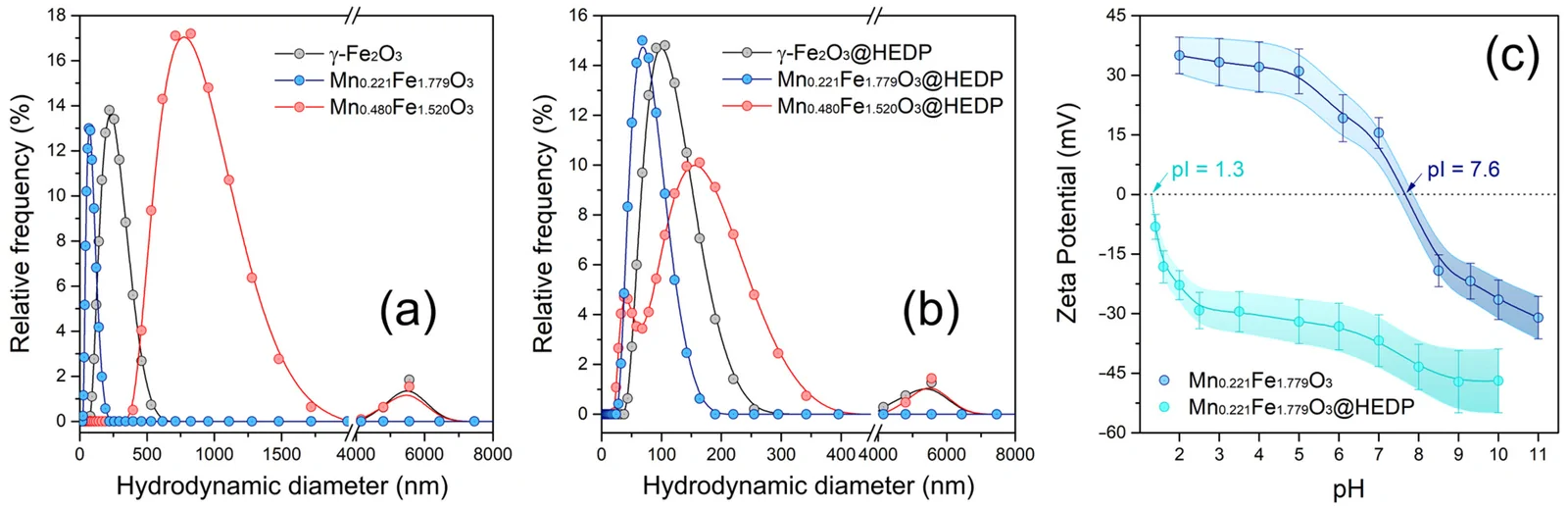
(**a**,**b**) Hydrodynamic diameter of bare and HEDP-coated Mn*_x_*Fe_2−*x*_O_3_ nanoparticles. (**c**) Zeta potential versus pH of bare and HEDP-coated Mn_0.221_Fe_1.779_O_3_.

Because colloidal stability strongly influences the in vivo behavior of nanoparticles, and may affect intratumoral distribution, radionuclide delivery, and magnetic performance, the colloidal properties of bare and HEDP-functionalized Mn*_x_*Fe_2−*x*_O_3_ nanoparticles were evaluated by dynamic light scattering (DLS) and zeta potential measurements. These analyses were performed to assess the effects of Mn substitution and HEDP functionalization on particle dispersion (Figure 2). DLS analysis showed a non-linear relationship between Mn content and hydrodynamic size (Figure 2a and Table 2). Moderate Mn substitution (*x* = 0.221) reduced the hydrodynamic diameter and produced a narrower size distribution than pure γ-Fe_2_O_3_. Increasing the Mn content to *x* = 0.480 had the opposite effect, with the average hydrodynamic diameter increasing to approximately 830 nm. The differences in hydrodynamic diameter among the bare nanoparticles likely reflect both differences in the organization of the multicore structures and their degree of association in suspension. This interpretation is qualitatively consistent with the TEM observations (Figure 1), which showed composition-dependent differences in nanoflower organization and more pronounced association of multicore units at the highest Mn content. The pronounced aggregation observed at the highest Mn content may be associated with changes in interparticle interactions. Magnetic dipole–dipole interactions can contribute to particle association in magnetic nanoparticle suspensions, although the present data do not allow their contribution to be distinguished from other colloidal interactions [23]. Together, the TEM and DLS observations suggest that Mn composition affects both the organization of the multicore structures and their collective behavior in suspension. The dramatic reduction in hydrodynamic diameter after HEDP functionalization of Mn_0.480_Fe_1.520_O_3_ (from ~830 nm to ~95 nm) further indicates that the very large hydrodynamic size of the bare Mn_0.480_Fe_1.520_O_3_ sample predominantly reflects secondary particle association rather than the dimensions of individual multicore units.

The effect of HEDP functionalization depended strongly on the initial colloidal state of the nanoparticles (Figure 2b). Only a marginal change in hydrodynamic diameter was observed for Mn_0.221_Fe_1.779_O_3_ (65 ± 13 nm to 63 ± 9 nm) (Figure S2), indicating a relatively well-dispersed colloidal state prior to surface modification. In contrast, HEDP substantially reduced the hydrodynamic diameter of γ-Fe_2_O_3_ (230 ± 70 nm to 100 ± 20 nm) and especially Mn_0.480_Fe_1.520_O_3_ (830 ± 170 nm to 95 ± 35 nm), indicating its effectiveness in suppressing secondary aggregation in formulations prone to interparticle association. Thus, the influence of HEDP became increasingly pronounced as the tendency toward aggregation increased, suggesting that surface engineering effectively counteracts particle association in aggregation-prone formulations. The HEDP coating also narrowed the particle size distribution of γ-Fe_2_O_3_ and Mn_0.221_Fe_1.779_O_3_ with Mn_0.221_Fe_1.779_O_3_@HEDP exhibiting the smallest hydrodynamic diameter (*D_h_* ≈ 63 nm) and the lowest polydispersity index (*PdI* = 0.135). In contrast, for Mn_0.480_Fe_1.520_O_3_, the marked decrease in hydrodynamic diameter after HEDP functionalization was accompanied by a higher *PdI* (0.382), indicating that the resulting dispersion remained comparatively heterogeneous despite the substantial reduction in secondary aggregation. This behavior is consistent with the strong affinity of phosphonate groups for iron oxide surfaces [22]. Once adsorbed, HEDP imparts a negative surface charge that increases electrostatic repulsion between neighboring nanoparticles and limits secondary aggregation [14,22]. Maintaining relatively small and well-dispersed nanoparticle assemblies may favor intratumoral transport and distribution and may also influence magnetic heating behavior. Accordingly, Mn_0.221_Fe_1.779_O_3_@HEDP was selected for subsequent radiolabeling and biological evaluation because it provided the most favorable balance between particle size, size distribution, and colloidal stability. The DLS results were further supported by zeta potential measurements (Figure 2c). HEDP functionalization shifted the isoelectric point (*pI*) of Mn_0.221_Fe_1.779_O_3_ from approximately pH 7.6 to pH 1.3, consistent with the presence of ionizable phosphonate groups at the nanoparticle surface. As a result, the zeta potential at physiological pH changed from approximately +4.9 mV to nearly −40 mV. This value exceeds the commonly used empirical threshold of approximately ±30 mV associated with strong electrostatic stabilization of colloidal dispersions, indicating substantial electrostatic repulsion at approximately physiological pH [24].

These electrostatic characteristics are fully consistent with the DLS measurements, indicating that HEDP adsorption effectively suppresses secondary particle aggregation and enhances electrostatic stabilization. The agreement between the DLS and zeta potential measurements supports the role of the phosphonate coating in improving colloidal dispersion. Together, these results show that HEDP plays a dual role within the nanoplatform: it stabilizes the nanoparticle suspension while simultaneously providing the phosphonate-rich surface required for efficient ^177^Lu coordination. More importantly, these findings suggest that compositional engineering of the magnetic core and surface engineering through HEDP functionalization play complementary roles, with both composition and surface chemistry contributing to the physicochemical properties required for subsequent radiolabeling and biological evaluation. The selected Mn_0.221_Fe_1.779_O_3_@HEDP formulation (NP2) was additionally assessed by visual inspection during storage in water at 4 °C. Photographs acquired at defined time points showed no visible sedimentation over the observation period (Figure S3). However, because DLS and zeta-potential measurements were performed in water, these results do not establish colloidal stability in biological media. Interactions with proteins and electrolytes may alter nanoparticle aggregation and consequently affect magnetic-heating performance and biodistribution in vivo. In addition, the DLS and zeta-potential measurements describe the dispersion state at the time of measurement and do not constitute a formal time-resolved colloidal stability study.

### 3.3. Magnetic Hyperthermia

**Figure 3:**
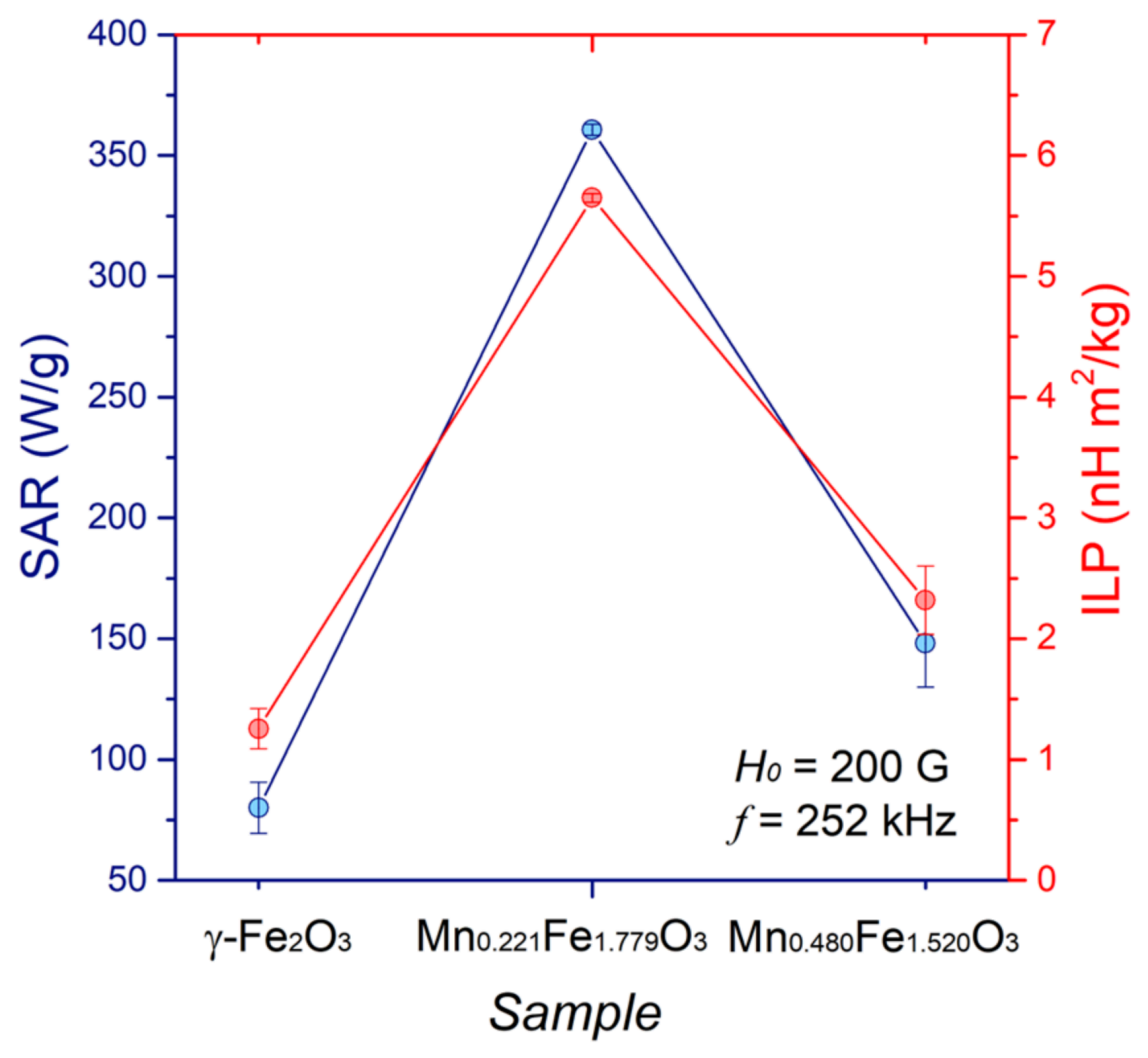
Specific absorption rate (SAR) and intrinsic loss power (ILP) for Mn*_x_*Fe_2−*x*_O_3_@HEDP.

To determine whether the observed differences in colloidal behavior translate into functional performance, the magnetic hyperthermia properties of all three HEDP-coated samples were evaluated. The HEDP-coated nanoparticles efficiently generated heat under an alternating magnetic field (*H*_0_ = 200 Oe, *f* = 252 kHz). Mn_0.221_Fe_1.779_O_3_@HEDP reached the therapeutic temperature of 42 °C in about 60 s (Figure S4). The *H* × *f* product (~4.0 × 10^9^ A m^−1^ s^−1^) was approximately equal to the Hergt–Dutz criterion (4.0 × 10^9^ A m^−1^ s^−1^) proposed for localized exposure of small body regions, indicating that the applied field conditions were within commonly accepted safety limits for magnetic hyperthermia [25]. Further Mn substitution beyond *x* = 0.221 did not improve the heating efficiency. Instead, the highest SAR and ILP values were obtained for Mn_0.221_Fe_1.779_O_3_@HEDP. This formulation reached an SAR of 360 ± 2 W g^−1^ and an ILP of 5.65 ± 0.04 nH m^2^/kg, whereas γ-Fe_2_O_3_@HEDP and Mn_0.480_Fe_1.520_O_3_@HEDP showed considerably lower values (Figure 3, Table 3). This non-monotonic dependence on Mn content is consistent with previous studies showing that the hyperthermic response of Mn-substituted iron oxides does not scale directly with Mn concentration, but may be influenced by cation distribution, magnetic anisotropy, particle organization, and interparticle interactions [18,26]. The colloidal characterization provides a probable explanation for this behavior. Notably, Mn_0.221_Fe_1.779_O_3_@HEDP combined the most favorable dispersion characteristics in water with the highest SAR and ILP values. In contrast, Mn_0.480_Fe_1.520_O_3_@HEDP formed large hydrodynamic aggregates. Such aggregates can reduce particle mobility and modify magnetic interactions between neighboring nanoparticles, thereby affecting magnetic relaxation and potentially lowering heat generation under an alternating magnetic field [27,28,29]. Importantly, magnetic interactions within multicore assemblies are not intrinsically harmful to hyperthermia; depending on their strength and organization, they may enhance or suppress heating. Excessive aggregation can alter this interaction regime and thereby shift the system away from conditions favorable for efficient energy dissipation [18,28]. Thus, excessive Mn incorporation does not necessarily improve magnetic heating because the beneficial effect of compositional tuning may be offset by aggregation-induced changes in collective magnetic behavior. Good colloidal stability alone does not fully explain the observed trend. Moderate Mn substitution may also modify the magnetic structure and effective anisotropy of the spinel lattice, thereby influencing magnetic relaxation and heating efficiency [18,26]. The highest heating efficiency is therefore likely associated with the combination of favorable magnetic properties and good colloidal dispersion rather than either factor alone [28]. These findings suggest that magnetic performance is governed by the interplay between intrinsic particle properties and their colloidal state, rather than only by Mn content. Because SAR depends on the applied magnetic-field amplitude, frequency, and experimental conditions, ILP was additionally calculated to normalize SAR with respect to frequency and field amplitude and to facilitate comparison of the heating efficiency of the investigated formulations. Both SAR and ILP identified Mn_0.221_Fe_1.779_O_3_@HEDP as the formulation with the highest magnetic-heating performance among the samples evaluated. The correspondence between the DLS and hyperthermia results suggests that moderate Mn substitution provides the best balance between magnetic functionality and colloidal stability. Accordingly, Mn_0.221_Fe_1.779_O_3_@HEDP was selected as the best sample for subsequent radiolabeling and in vivo evaluation because it exhibited the most favorable combination of magnetic performance and colloidal behavior, whereas γ-Fe_2_O_3_@HEDP was retained as a reference formulation to assess the contribution of Mn substitution to the overall performance of the nanoplatform. Collectively, these results suggest that optimization of Mn composition should consider both intrinsic magnetic properties and colloidal stability rather than magnetic performance alone.

Although the present study focuses exclusively on localized radionuclide therapy, magnetic hyperthermia was evaluated as an additional functional characteristic of the engineered nanoplatform. No AMF was applied in vivo; therefore, combined treatment and potential synergy were not evaluated in the present study. The observed heating performance of the optimized sample highlights the potential of these HEDP-functionalized magnetic nanoparticles for future multimodal therapeutic strategies combining magnetic hyperthermia with localized radionuclide therapy.

### 3.4. Radiochemical Characterization of [^177^Lu]Lu-NP1 and [^177^Lu]Lu-NP2

#### 3.4.1. Radiolabeling

To evaluate the radiopharmaceutical performance of the optimized magnetic nanoplatform, the lead formulation, Mn_0.221_Fe_1.779_O_3_@HEDP (NP2), and the reference γ-Fe_2_O_3_@HEDP sample (NP1) were radiolabeled with [^177^Lu]Lu^3+^ using a direct radiolabeling approach. Radiolabeling yields of 86.6 ± 2.1% and 92.3 ± 2.5% were obtained for NP1 and NP2, respectively. Both formulations showed efficient radiolabeling, indicating that moderate Mn substitution did not impair the radiometal-coordinating function of the HEDP layer. The ITLC results were further supported by the centrifugation/gamma-counting analysis described in Section 2.5, which confirmed that the majority of the measured radioactivity remained associated with the nanoparticle fraction. These findings indicate that HEDP surface functionalization provides robust radiolabeling capability regardless of Mn incorporation, whereas Mn compositional engineering improves the physicochemical and magnetic characteristics of the nanoplatform without compromising its radiolabeling performance. Consequently, the γ-Fe_2_O_3_@HEDP reference formulation serves as an appropriate benchmark for assessing the additional contribution of Mn substitution to the overall performance of the nanoplatform independently of radiolabeling efficiency.

#### 3.4.2. In Vitro Stability of [^177^Lu]Lu-NP1 and [^177^Lu]Lu-NP2

**Figure 4:**
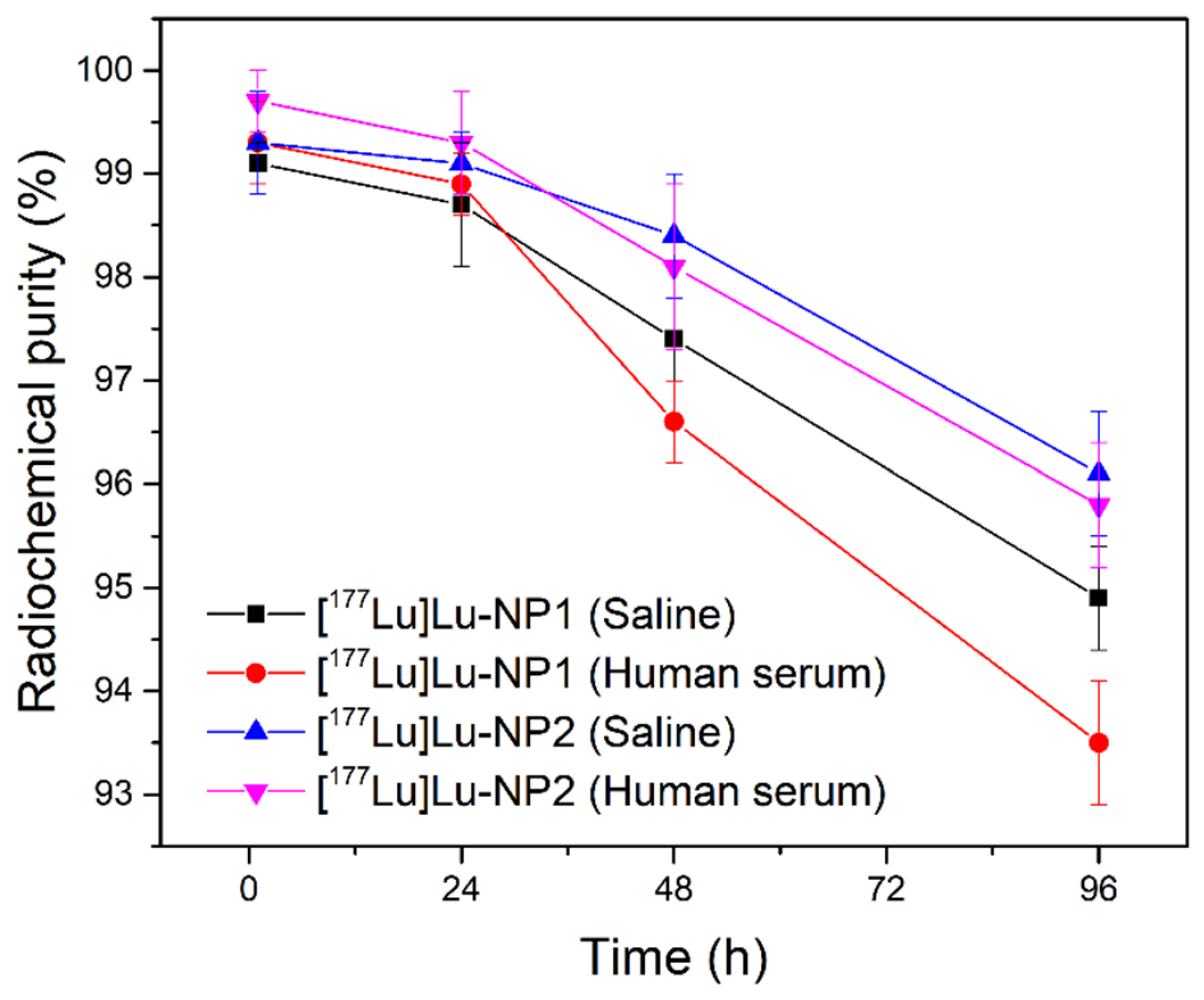
In vitro stability of [^177^Lu]Lu-NP1 and [^177^Lu]Lu-NP2 in saline and human serum at 37 °C over 96 h. Radiochemical purity determined by radio-TLC is expressed as mean ± SD.

Since efficient radiolabeling alone does not guarantee the stability of a radiopharmaceutical following intratumoral administration, the radiochemical stability of both constructs was evaluated under physiologically relevant conditions. To completely remove any unbound [^177^Lu]Lu^3+^ for subsequent stability and biodistribution studies, [^177^Lu]Lu-NP1 and [^177^Lu]Lu-NP2 were separated by centrifugation, supernatants were discarded and nanoparticles were resuspended in different media. For the in vitro stability study, [^177^Lu]Lu-NP1 and [^177^Lu]Lu-NP2 were incubated at 37 °C in saline and human serum, and radiochemical purity was monitored by radio-TLC after 1, 24, 48, and 96 h. Both formulations remained highly stable throughout the entire incubation period (Figure 4). After 96 h, the released radioactivity remained low, amounting to 4.2 ± 0.5% and 5.8 ± 0.8% for [^177^Lu]Lu-NP1 in saline and human serum, respectively, compared with 3.2 ± 0.4% and 3.9 ± 0.8% for [^177^Lu]Lu-NP2 under the same conditions. Accordingly, the radiochemical purity of both formulations remained above 94% after 96 h of incubation. High radiochemical stability is particularly important for localized radionuclide therapy, because premature radionuclide release could reduce radiation delivery to the tumor while increasing radionuclide redistribution and radiation exposure to healthy tissues [30]. The stability observed for both formulations indicates that HEDP surface functionalization provides sufficiently stable coordination of ^177^Lu under physiologically relevant conditions. This interpretation is supported by the recent work of Sitarica et al., who combined electrochemical measurements with density functional theory (DFT) calculations to investigate the coordination chemistry of HEDP with trivalent lanthanide ions [13]. That study identified a tridentate coordination mode, in which HEDP binds the metal ion through oxygen atoms from both phosphonate groups, as the energetically preferred structure. Similar DFT calculations performed for Lu^3+^ predicted the same favorable coordination geometry [13], providing a molecular basis that is consistent with the high radiochemical stability observed in the present study.

These results demonstrate that HEDP surface functionalization enables stable radionuclide coordination regardless of Mn incorporation, resulting in comparable radiochemical stability for both tested formulations. Together with the radiolabeling data, these results indicate that Mn compositional engineering enhances the physicochemical and magnetic performance of the nanoplatform without compromising the stability of its radiopharmaceutical component, thereby supporting its further evaluation in vivo.

### 3.5. Biodistribution Studies of [^177^Lu]Lu-NP1 and [^177^Lu]Lu-NP2

#### 3.5.1. In Vivo Whole-Body Imaging

**Figure 5:**
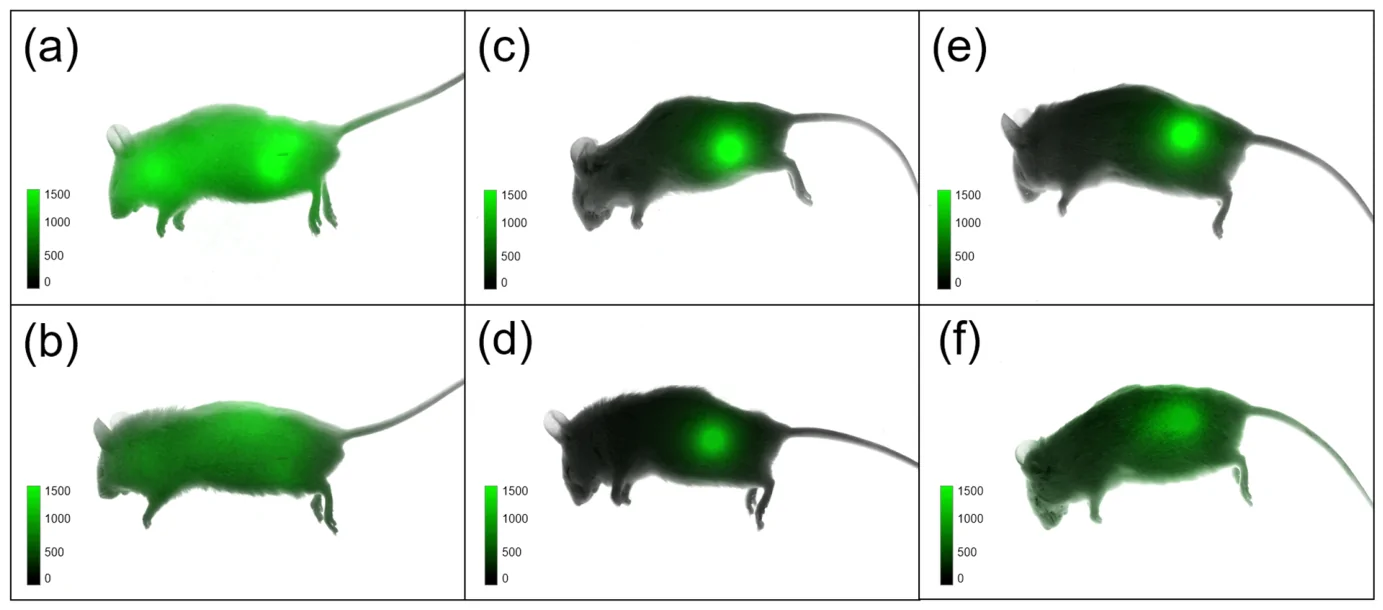
Qualitative whole-body images of individual 4T1 tumor-bearing mice acquired on days 5 and 14 after intratumoral administration of (**a**,**b**) free [^177^Lu]LuCl_3_, (**c**,**d**) [^177^Lu]Lu-NP1, and (**e**,**f**) [^177^Lu]Lu-NP2. Panels (**a**,**c**,**e**) show images acquired on day 5, whereas panels (**b**,**d**,**f**) show images acquired on day 14.

Having established efficient radiolabeling and high radiochemical stability, the retention of radioactivity at the site of intratumoral administration was qualitatively evaluated by whole-body imaging at two time points in mice bearing 4T1 tumors. Imaging was performed on one mouse from each group receiving free [^177^Lu]LuCl_3_, [^177^Lu]Lu-NP1, or [^177^Lu]Lu-NP2. The same animals were imaged on days 5 and 14 after intratumoral administration, and the resulting qualitative images are presented in Figure 5.

For both nanoparticle formulations, the images showed persistent tumor-associated radioactivity on days 5 and 14, consistent with prolonged retention of the radiolabeled nanoparticles following intratumoral administration. In contrast, the mouse receiving free [^177^Lu]LuCl_3_ showed a markedly more widespread whole-body distribution at both imaging time points, indicating redistribution of the unbound radionuclide away from the injection site.

These qualitative findings indicate that the HEDP-functionalized nanoparticles retained ^177^Lu at the tumor site, whereas free [^177^Lu]LuCl_3_ underwent redistribution to non-target tissues. Similar prolonged retention following intratumoral administration has been reported for other ^177^Lu-labeled nanocarriers, including gold nanostars that remained detectable in tumors for up to 15 days [31,32]. The importance of formulation-dependent retention was also demonstrated by Cui et al., who incorporated chelator-free radiolabeled iron–tannic acid nanoparticles into an injectable hydrogel to prolong radionuclide retention within the tumor [33]. Although retention in their system was additionally supported by the hydrogel matrix, their findings reinforce the general principle that immobilization of a therapeutic radionuclide at the injection site can sustain local irradiation and limit systemic redistribution. NP1 and NP2 showed similar qualitative localization patterns, suggesting that partial substitution of Fe by Mn did not compromise radionuclide retention following intratumoral administration.

Whole-body imaging, however, provides qualitative information on the overall localization of radioactivity and does not reveal its spatial distribution throughout the tumor tissue. Consequently, it cannot distinguish between homogeneous intratumoral distribution and localization around the injection site. Addressing this limitation will require future studies combining autoradiography with histological analysis, as recently demonstrated by Sahota et al., whose integrated imaging workflow enables high-resolution spatial mapping of radionuclide distribution within tissue sections [34].

#### 3.5.2. Ex Vivo Biodistribution Studies

**Figure 6:**
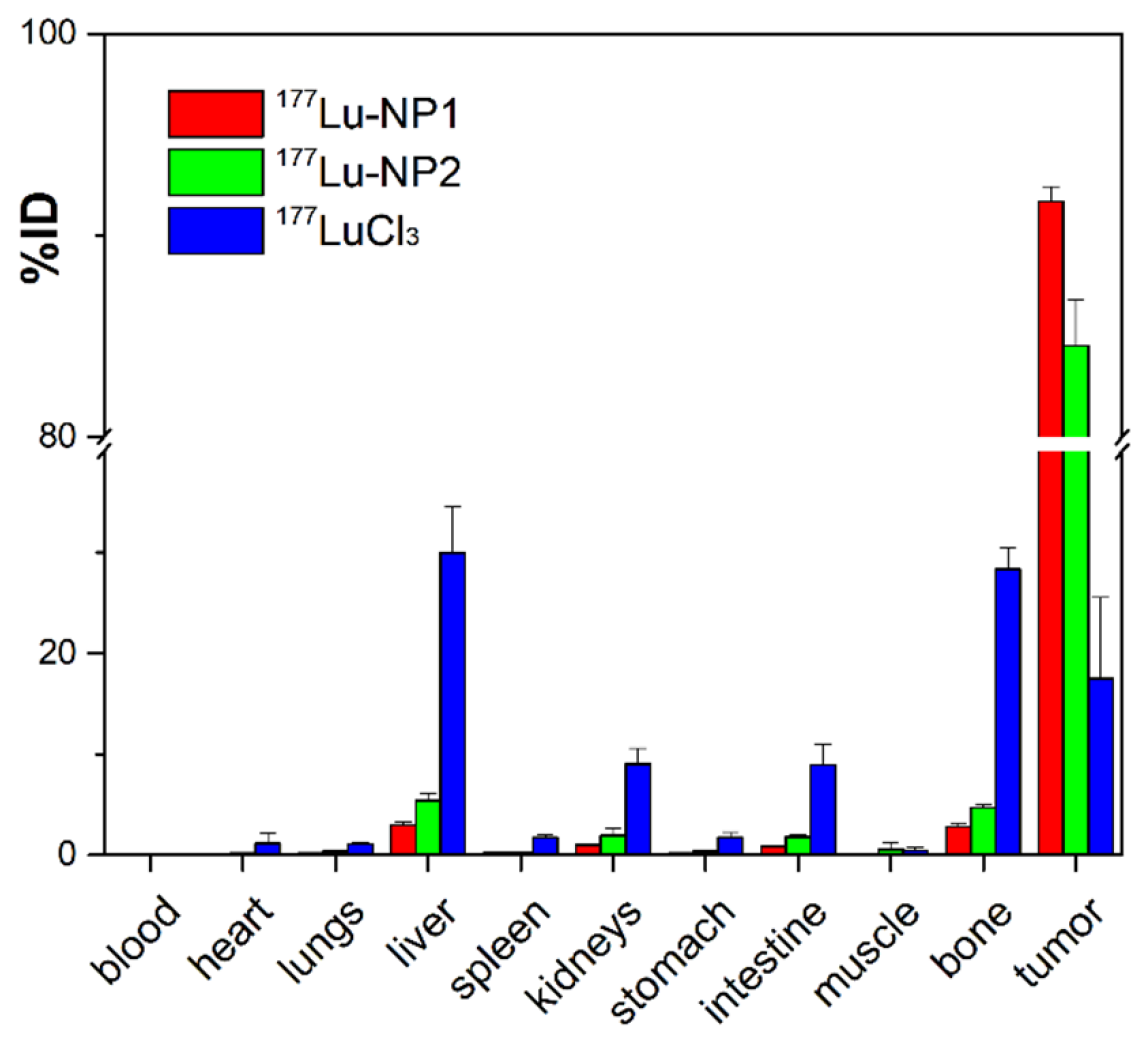
Ex vivo biodistribution of [^177^Lu]Lu-NP1, [^177^Lu]Lu-NP2, and free [^177^Lu]LuCl_3_ in tumors and major organs 14 days after intratumoral administration (7.4 MBq in 50 μL). Data are presented as mean ± SD (n = 6 per group).

The whole-body imaging findings were further supported by quantitative ex vivo biodistribution analysis using γ-counting, performed 14 days after intratumoral administration. Tumor-associated radioactivity accounted for 91.68 ± 0.73%ID in mice treated with [^177^Lu]Lu-NP1 and 84.53 ± 2.26%ID in those treated with [^177^Lu]Lu-NP2. In contrast, only low levels of radioactivity were detected in the analyzed non-target organs (Figure 6).

In contrast, mice receiving free [^177^Lu]LuCl_3_ showed substantially lower tumor-associated radioactivity (17.49 ± 8.06%ID) and higher radioactivity in the bone (28.35 ± 2.19%ID) and liver (29.97 ± 4.63%ID). This distribution is consistent with the systemic redistribution of non-nanoparticle-bound ^177^Lu and the known accumulation of radiolanthanides in the liver and bone [35]. These results indicate that the HEDP-functionalized nanoparticle formulations enabled high tumor-associated retention of ^177^Lu, with limited redistribution to the analyzed non-target tissues. Both NP1 and NP2 exhibited comparable biodistribution profiles, characterized by predominant tumor-associated radioactivity and relatively low radioactivity in the analyzed non-target organs. Under the evaluated conditions, partial substitution of Fe by Mn did not substantially affect the biodistribution of the radiolabeled nanoplatform, suggesting that Mn incorporation did not compromise its intratumoral retention.

### 3.6. Therapeutic Efficacy of [^177^Lu]Lu-NP1 and [^177^Lu]Lu-NP2

**Figure 7:**
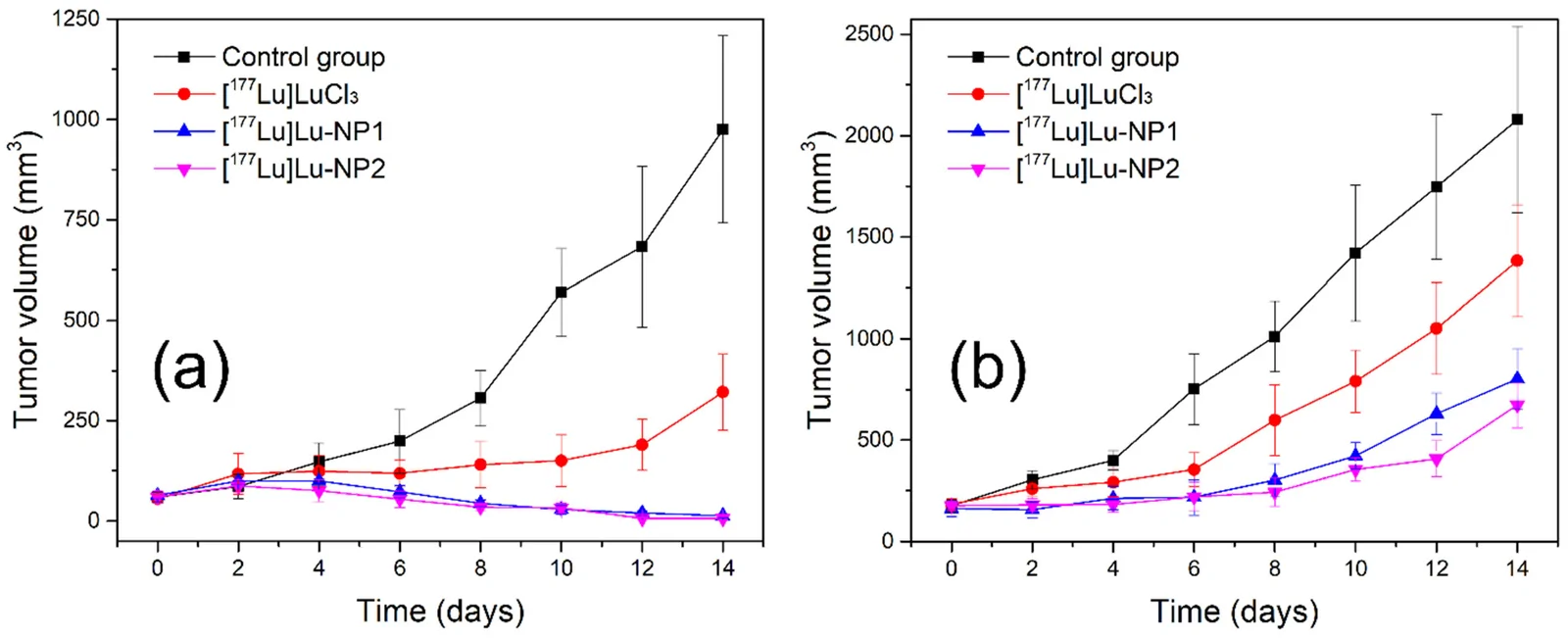
Tumor growth curves in BALB/c mice bearing 4T1 tumors following intratumoral administration of saline, free [^177^Lu]LuCl_3_ or [^177^Lu]Lu-NP1, or [^177^Lu]Lu-NP2. (**a**) Mice with small initial tumor volumes (45–75 mm^3^). (**b**) Mice with large initial tumor volumes (150–200 mm^3^). Data are presented as mean ± SD (n = 6 per group).

The therapeutic efficacy of [^177^Lu]Lu-NP1 and [^177^Lu]Lu-NP2 in relation to free [^177^Lu]LuCl_3_, was evaluated in mice bearing small (45–75 mm^3^) or large (150–200 mm^3^) 4T1 tumors (Figure 7).

**Figure 8:**
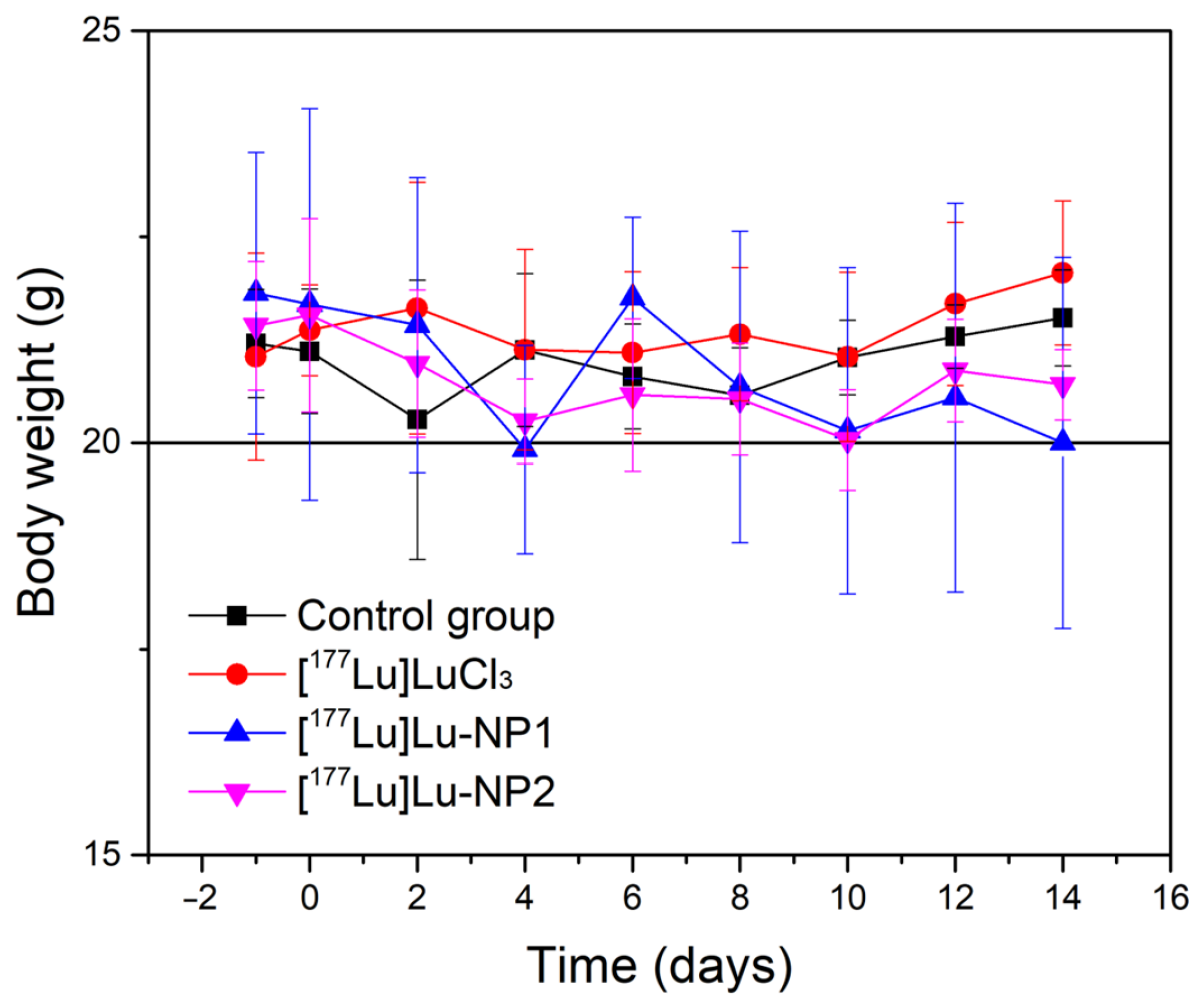
Body weight changes in BALB/c mice bearing subcutaneous 4T1 tumors during the 14-day treatment period. Data are presented as mean ± SD (n = 6 per group).

Both nanoparticle formulations showed marked inhibition of tumor growth relative to the saline-treated control group. The therapeutic response was more pronounced in animals with smaller tumors, in which marked tumor growth inhibition and partial tumor regression were observed, whereas treatment of larger tumors was associated with a less pronounced inhibition of tumor growth. Free [^177^Lu]LuCl_3_ reduced tumor growth compared with the saline control, but the antitumor effect was less pronounced than that observed with the nanoparticle-based formulations. No marked changes in body weight were observed in any treatment group throughout the study period (Figure 8), indicating that none of the treatments resulted in detectable body-weight loss during the 14-day observation period. However, body-weight monitoring alone does not constitute a comprehensive safety assessment, and the absence of hematological, histopathological, and Mn-accumulation data precludes conclusions regarding systemic or Mn-related toxicity. Similar therapeutic effects of radiolabeled nanoparticles have been reported previously in experimental 4T1 breast cancer and other murine tumor models [31,36]. NP1 and NP2 showed similar tumor-growth profiles, suggesting that moderate Mn substitution did not measurably compromise therapeutic performance under the evaluated conditions. Both formulations showed persistent tumor-associated radioactivity at the evaluated imaging time points and high tumor-associated radioactivity on day 14. Nevertheless, the magnitude of the therapeutic response appeared to be associated with initial tumor volume.

Because the same administered activity was used for all animals, smaller tumors may have received higher activity concentrations, assuming comparable radionuclide retention, thereby potentially resulting in higher absorbed doses in the tumor. Previous experimental studies have demonstrated that tumor size can influence radionuclide uptake and intratumoral absorbed dose, thereby affecting the resulting therapeutic efficacy [37]. In addition, structural heterogeneity, elevated interstitial pressure, a dense extracellular matrix, and necrotic regions may limit the penetration and spatial distribution of locally administered nanoparticles, particularly in larger tumors [4,38]. Such barriers may result in high tumor-associated radioactivity while leaving individual tumor regions insufficiently exposed to the therapeutic radionuclide [39,40].

Overall, these findings indicate that prolonged intratumoral retention is an important prerequisite for effective localized radionuclide therapy but is not, by itself, sufficient to determine therapeutic outcome. The proposed relationships among initial tumor size, intratumoral radionuclide distribution, absorbed dose, and therapeutic response require further experimental validation. The 14-day observation period permitted assessment of short-term tumor-growth response but did not allow evaluation of durable tumor control, tumor recurrence, or overall survival. Longer follow-up will be required to determine whether the observed tumor-growth inhibition translates into sustained therapeutic benefit.

## 4. Conclusions

This study demonstrates that compositional and surface engineering can be combined to produce HEDP-functionalized magnetic nanoparticles with both magnetic-heating and ^177^Lu-labeling functionalities. Among the tested compositions, moderate Mn substitution improved the magnetic-heating performance and aqueous dispersion characteristics under the evaluated experimental conditions, whereas HEDP functionalization enabled efficient direct radiolabeling with ^177^Lu followed by high in vitro radiochemical stability. Under the evaluated conditions, Mn substitution did not markedly affect radiolabeling efficiency, radiochemical stability, the overall biodistribution pattern, or the therapeutic performance of the HEDP-functionalized nanoparticles.

Following intratumoral administration, qualitative whole-body imaging showed persistent tumor-associated radioactivity for both radiolabeled nanoparticle formulations at the evaluated imaging time points, while quantitative analysis on day 14 showed high tumor-associated radioactivity and low radioactivity in the analyzed non-target organs. Both formulations also inhibited tumor growth during the 14-day observation period, with a more pronounced response and partial tumor regression observed in animals with smaller initial tumors. The different responses observed in the predefined tumor-size cohorts suggest a possible association between initial tumor volume and therapeutic response. Differences in activity concentration and spatial distribution of radioactivity within the tumor may also contribute, although direct dosimetry and spatial mapping of radioactivity will be required to evaluate these possibilities. The present therapeutic findings are limited to the 14-day observation period and do not establish durable tumor control or survival benefit.

Overall, moderate Mn substitution and HEDP functionalization represent complementary design strategies for the development of radiopharmaceuticals based on magnetic nanoparticles intended for localized radionuclide therapy. The resulting nanoplatform combines favorable magnetic-heating and radiopharmaceutical properties and provides a basis for future preclinical studies directly evaluating the combination of localized radionuclide therapy and magnetic hyperthermia within the same experimental model.

## Figures and Tables

**Table 1:** Initial precursor stoichiometry, composition determined by ICP-OES, Mn incorporation efficiency, and structural parameters of the synthesized Mn_x_Fe_2−x_O_3_ nanoparticles.

Initial Precursor Composition	Composition Determined by ICP-OES	Mn Incorporation Efficiency (%)	Crystallite Size (nm)
γ-Fe_2_O_3_	γ-Fe_2_O_3_	–	20.4 ± 0.8
Mn_0.333_Fe_1.667_O_3_	Mn_0.221_Fe_1.779_O_3_	66.4	15.2 ± 0.7
Mn_0.666_Fe_1.334_O_3_	Mn_0.480_Fe_1.520_O_3_	72.1	15 ± 3

**Table 2:** Colloidal properties of Mn*_x_*Fe_2−*x*_O_3_.

Sample	Hydrodynamic Diameter (nm)	PdI	Coated Sample	Hydrodynamic Diameter (nm)	PdI
γ-Fe_2_O_3_	230 ± 70	0.291	γ-Fe_2_O_3_ @HEDP	100 ± 20	0.230
Mn_0.221_Fe_1.779_O_3_	65 ± 13	0.197	Mn_0.221_Fe_1.779_O_3_@HEDP	63 ± 9	0.135
Mn_0.480_Fe_1.520_O_3_	830 ± 170	0.203	Mn_0.480_Fe_1.520_O_3_@HEDP	95 ± 35	0.382

**Table 3:** Magnetic hyperthermia efficacy of Mn*_x_*Fe_2−*x*_O_3_@HEDP.

Sample	SAR (W/g)	ILP (nH m^2^/kg)
γ-Fe_2_O_3_@HEDP	80 ± 10	1.3 ± 0.2
Mn_0.221_Fe_1.779_O_3_@HEDP	360 ± 2	5.65 ± 0.04
Mn_0.480_Fe_1.520_O_3_@HEDP	150 ± 20	2.3 ± 0.3

## Data Availability

The original contributions presented in this study are included in the article. Further inquiries can be directed to the corresponding author.

## Supplementary Materials

The following supporting information can be downloaded at https://www.mdpi.com/article/10.3390/pharmaceutics18101324/s1: Figure S1: FTIR spectra of Mn*_x_*Fe_2−x_O_3_ and Mn*_x_*Fe_2−*x*_O_3_@HEDP samples; Figure S2: Hydrodynamic diameters of Mn*_x_*Fe_2−*x*_O_3_ and Mn*_x_*Fe_2−*x*_O_3_@HEDP samples; Figure S3. Photographs of the Mn_0.221_Fe_1.779_O_3_@HEDP suspension (NP2) during storage in water at 4 °C, acquired at the indicated time points; Figure S4: Calorimetric curves of HEDP-coated γ-Fe_2_O_3_ (a), Mn_0.221_Fe_1.779_O_3_@HEDP (b) and Mn_0.480_Fe_1.520_O_3_@HEDP (c) nanoparticles.
