# Supplementary material for: 177Lu-Labeled HEDP-Functionalized Manganese-Substituted Maghemite Nanocarriers for Localized Radionuclide Therapy
Source: Pharmaceutics. 2026 Oct 19;18(10):1324. doi: 10.3390/pharmaceutics18101324 (PMC13708146; doi:10.3390/pharmaceutics18101324)
Supplement: Supplementary file 1 [file pharmaceutics-18-01324-s001.zip › pharmaceutics-4571715-supplementary.pdf]

Supplementary materials

^{177}Lu-Labeled HEDP-Functionalized Manganese-Substituted Maghemite Nanocarriers for Localized Radionuclide Therapy

Dragana Stanković, Magdalena Radović, Pavle Sitarica, Miloš Marić, Aleksandar Vukadinović, Miloš Ognjanović, Marija Mirković, Drina Janković, Mirjana Milić, Bratislav Antić and Sanja Vranješ Đurić *

VINČA Institute of Nuclear Sciences, National Institute of the Republic of Serbia, University of Belgrade, P.O. Box 522 Belgrade, Serbia

* Correspondence: sanjav@vin.bg.ac.rs

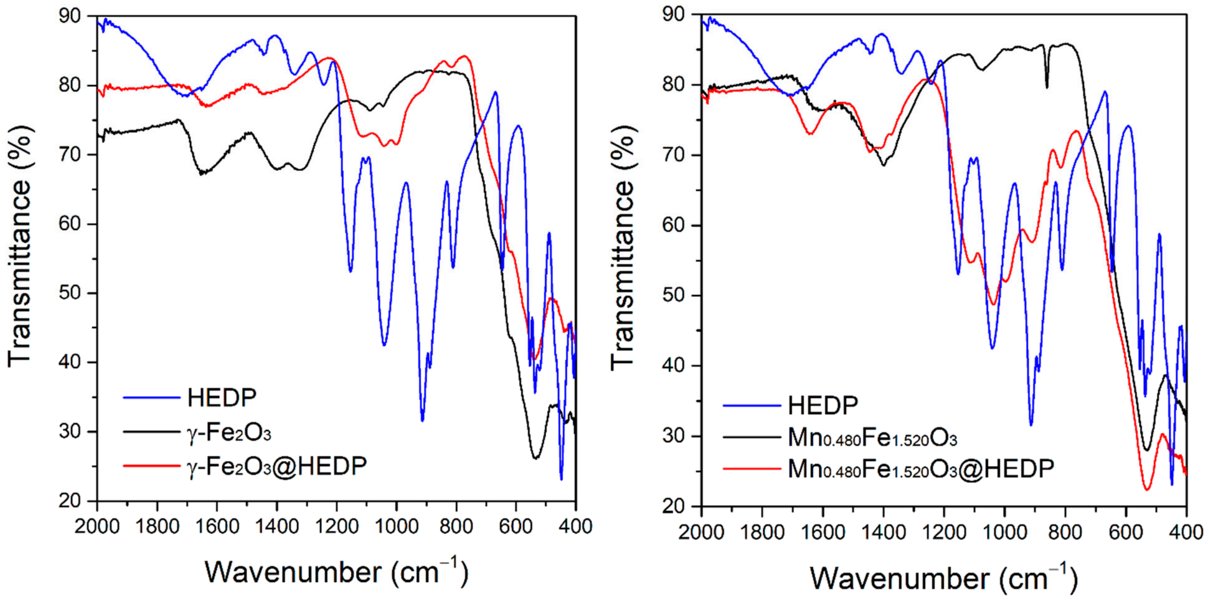

Figure S1. FTIR spectra of $Mn_xFe_{2-x}O_3$ and $Mn_xFe_{2-x}O_3$@HEDP samples.

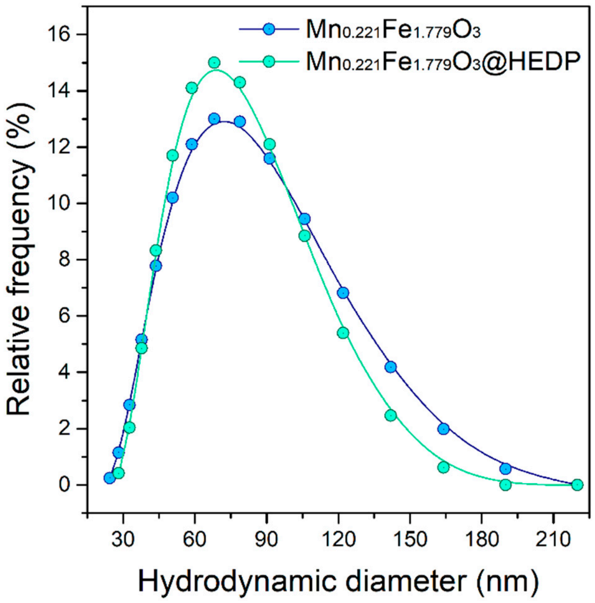

Figure S2. Hydrodynamic diameters of $Mn_xFe_{2-x}O_3$ and $Mn_xFe_{2-x}O_3$@HEDP samples.

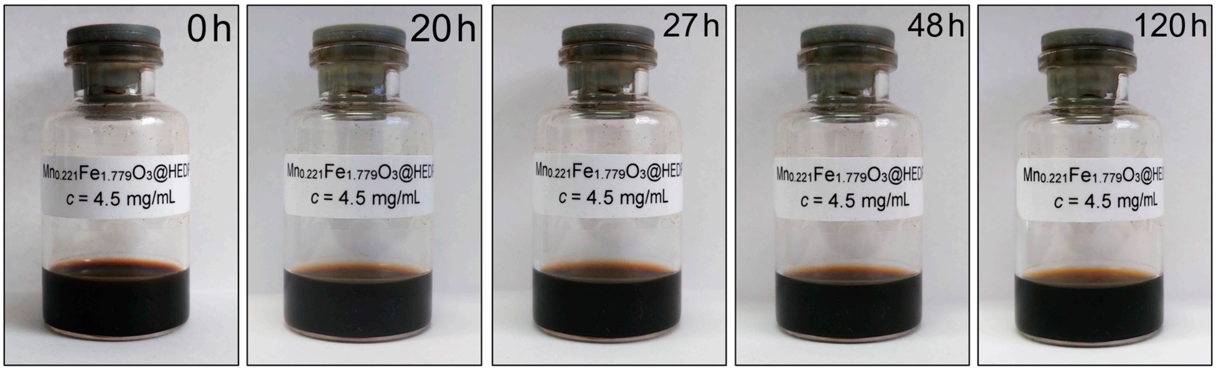

Figure S3. Photographs of the $Mn_{0.221}Fe_{1.779}O_3$@HEDP suspension (NP2) during storage in water at 4 °C, acquired at the indicated time points.

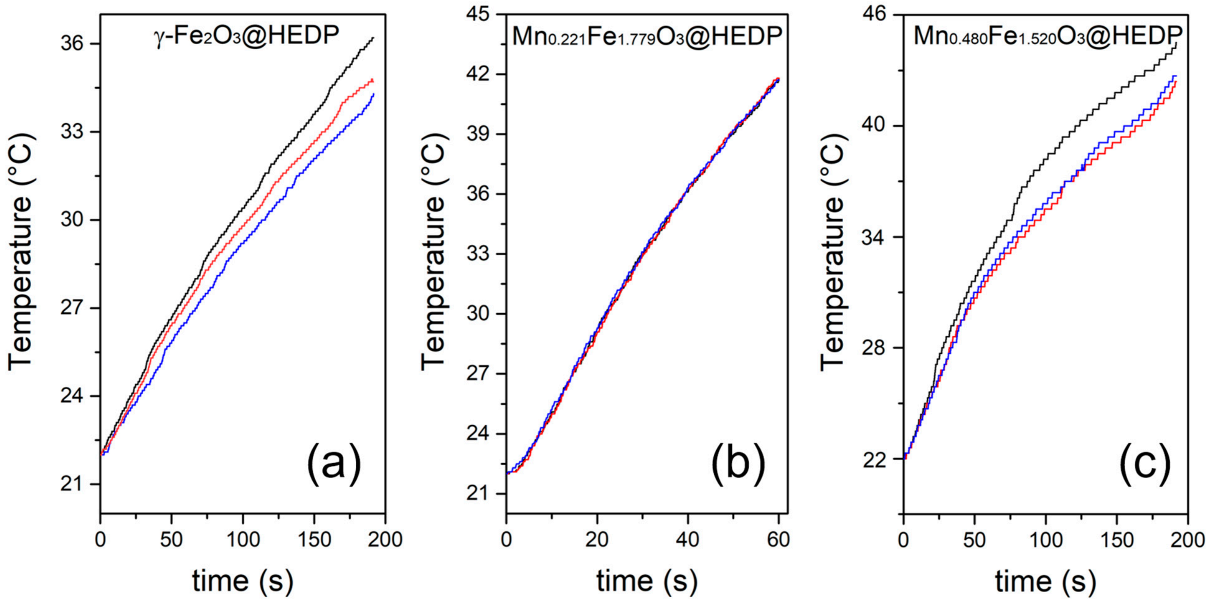

Figure S4. Calorimetric curves of HEDP-coated γ-Fe_2O_3 (a), $Mn_{0.221}Fe_{1.779}O_3$@HEDP (b) and $Mn_{0.480}Fe_{1.520}O_3$@HEDP (c) nanoparticles.
